# Biomechanical research of medial femoral circumflex vascularized bone-grafting in the treatment of early-to-mid osteonecrosis of the femoral head: a finite element analysis

**DOI:** 10.1186/s13018-022-03335-7

**Published:** 2022-10-04

**Authors:** Yingkai Zhang, Xinyuan Wang, Chang Jiang, Bingxuan Hua, Zuoqin Yan

**Affiliations:** 1 Department of Orthopaedic Surgery, Zhongshan Hospital Fudan University, Fenglin Road 180, Shanghai City, 200032 People’s Republic of China; 2 Department of Orthopaedic Surgery, Jinshan Hospital of Fudan University, Shanghai City, 201508 People’s Republic of China

**Keywords:** Osteonecrosis of the femoral head, Hip preservation therapy, Finite element analysis, Vascularized bone transplantation

## Abstract

**Purpose:**

Hip preservation therapy of early ONFH (Osteonecrosis of the femoral head) has emerged as one of the hot areas of research. We have optimized the procedure of traditional MFCVBG (medial femoral circumflex vascularized bone grafting) by using specialized surgical tools and used the finite element analysis to guide the implantation position of the bone flap during surgery and validate the biological mechanical stability of the modified MFCVBG.

**Methods:**

This study was based on the data of a male patient with left hip (ARCO stage IIB, JIC type C) hormonal ONFH. Harris score (HHS), anteroposterior and lateral hip radiographs, frog position hip radiographs and SPECT/CT of femoral head flow imaging were performed postoperatively to evaluate clinical efficacy. The patient’s CT data were used to establish upper femur finite element model of the normal group, osteonecrosis group and postoperative group, respectively. The force on the femoral structure of each group was analyzed under four different loads in the gait cycle of 0.5 times the body weight (0.5 G, standing on two feet), 2.75 G (standing on one foot), 4 G (walking with the middle foot on the ground) and 7 G (walking with the toe off the ground) to validate the biological mechanical stability of the modified MFCVBG, predict femoral head collapse risk, simulate of the different healing conditions of postoperative bone flap, and analyze the postoperative effect of non-ideal surgical model.

**Results:**

According to the follow-up results, the bone flap and the inner wall of decompression channel healed well, no osteonecrosis progression, no local collapse or micro-fracture occurred in the femoral head, and the articular surface was intact and the necrosis was well repaired. According to the result of the finite element analysis, compared with the osteonecrosis group, the overall stress and displacement peak of the upper femur and the cortical bone stress peak of the femoral head in the postoperative group and normal group were significantly reducing; modified MFCVBG can significantly improve the biomechanical stability of necrotic femoral head and reduce the risk of femoral head collapse; there was no obvious abnormal stress distribution in the greater trochanter and intertrochanter region after the flap was removed; the bone flap of the complete removal of necrotic focus + long bone flap group was directly placed at the bottom of the decompression passage, and the bone flap cortical bone can provide substantial mechanical support; in theory, patients can try to reduce the load with crutches or walking aids and carry out appropriate flat activities to effectively promote the early postoperative recovery.

**Conclusions:**

The modified MFCVBG resulted in good efficacy, safety and feasibility. The necrotic focus should be completely removed during the operation, and the long bone flap should be placed directly under the subchondral bone. For patients with better bone healing ability, a more positive attitude can be taken to promote early postoperative weight-bearing.

## Introduction

ONFH (Osteonecrosis of the femoral head) is a common refractory disease found in orthopedics. Trauma, long-term use of large doses of glucocorticoids, excessive alcohol intake and skeletally immature have been identified as most common risk factors [1, 2]. Currently, non-traumatic ONFH patients in China are being diagnosed at younger age [3]. For young patients with ONFH, early acceptance of THA (Total hip arthroplasty) often can result in increase in the possibility of undergoing revision surgery [4]. Therefore, hip preservation therapy of ONFH has emerged as one of the hot areas of research.

In the 1990s, Chen et al*.* [5] first proposed the application of surgical method based on using the greater trochanter bone flap with the deep branch of the medial femoral circumflex vessel through the posterior approach and inserting it through the posterior femoral head–neck junction area. As ONFH often occurs in the anterior side of the femoral head, the posterior approach decompression channel has been found to be more convenient for the complete removal of the necrotic bone tissues and providing mechanical support to the necrotic area. Therefore, it possesses certain advantages in technology. However, due to the limitations of surgical design, surgical tools and technology, the original surgical method has been found to be relatively difficult to operate and the effect might not be ideal: (1) The necrotic bone could not be removed completely due to the limitation of surgical tools; (2) only bone flap implantation is difficult to effectively support early postoperative activities; and (3) because the direction of core decompression was limited, the direction of bone flap implantation could not be determined, thus affecting the mechanical stability and recovery effect. We have advanced MFCVBG (medial femoral circumflex vascularized bone grafting) and established a standardized operation process for the treatment of ONFH in the peri-collapse period of the modified MFCVBG, to significantly improve the simplicity and refinement of the operation, and increase the effect of the surgical treatment. In this study, the 3D finite element method was used to analyze the mechanical impact of normal, necrotic and after modified MFCVBG surgery upper femur model, to guide the bone flap implantation position and validate the supporting effect of the modified MFCVBG on the necrotic surface of the femoral head from the perspective of biomechanics.

## Materials and methods

### ONFH staging and typing

In this study, ONFH staging refers to the staging method based on 2019 version of ARCO staging for the different necrotic areas [6]. The classification used in this study was derived from the classification of ONFH Japanese (Osteonecrosis) Investigation Committee (JIC) [7].

### Analysis of a typical case with ONFH

This study was based on the data of a patient with left hip (ARCO stage IIB, JIC type C) before and after operation of a male patient with bilateral hormonal ONFH, aged 22 years, 1.75 m in height and 72 kg in weight. The Harris score (HSS) of the patient is 71 points. This study was approved by the ethics committee (B2019-135R).

#### Surgical procedures

**Fig. 1:**
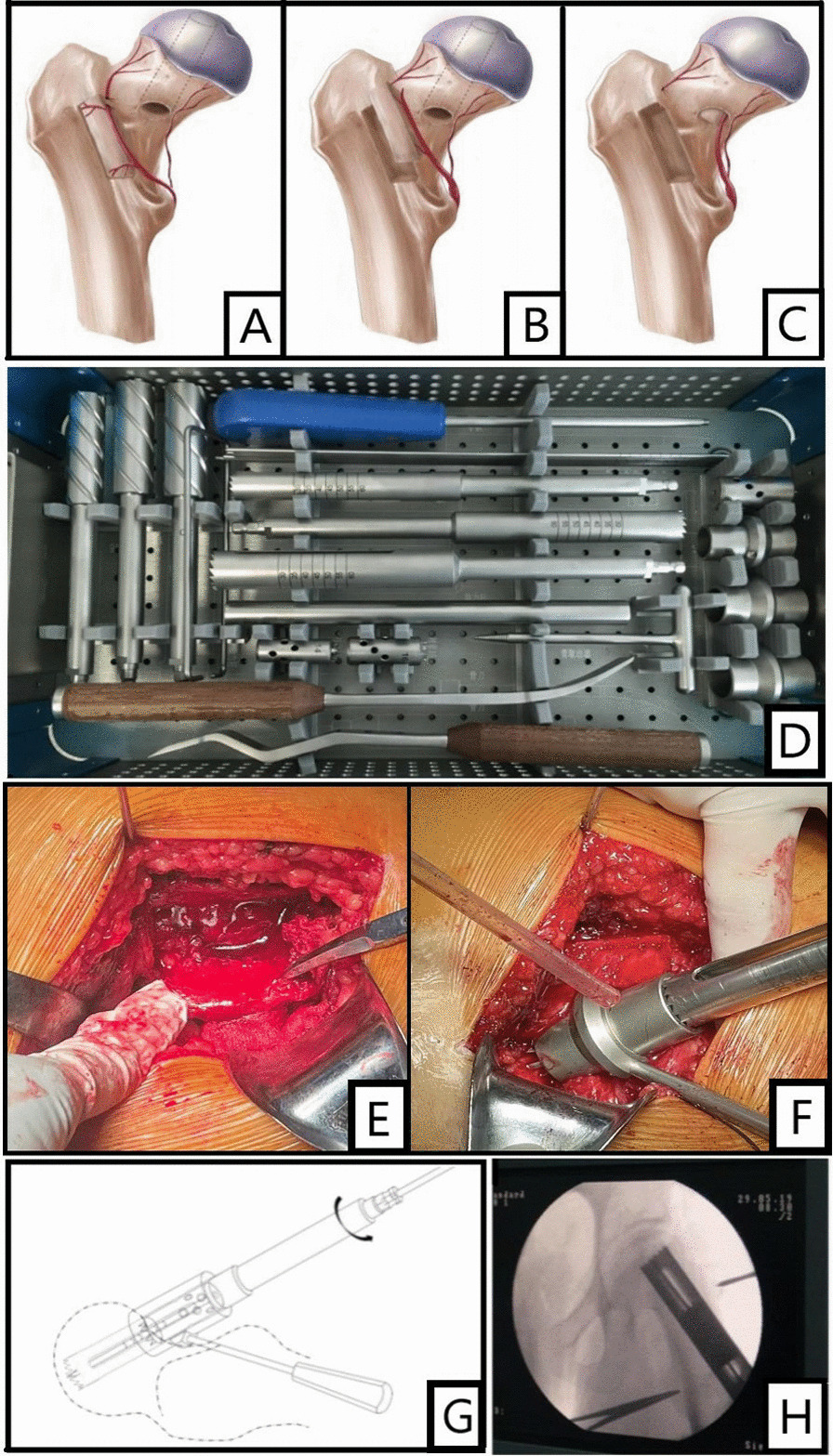
**A**–**C** Surgical steps of the improved MFCVBG; **D** the special surgical tool of modified MFCVBG; **E**–**F** the vascularized bone flap was cut during the operation; **G** schematic diagram of surgery; **H** intraoperative fluoroscopy: drilling pressure through large diameter trephine

The 90-degree lateral decubitus position was used during the operation. The strategy was the posterolateral approach of the traditional hip joint. The posterolateral curved incision of the hip was cut layer by layer. The gluteus maximus muscle was first passively separated, then the greater trochanter bursa was removed, and the hip joint was rotated inward. The upper and lower gemellus muscle was exposed, followed by dissection and exposure of the quadratus femoris. Thereafter, part of the quadratus femoris was cut, which separated and exposed the trunk and branches of the deep branch of the medial circumflex femoral artery. After the trunk was found, it was traced to the outside along its course. It was observed that there were branches leading to the greater trochanter and intertrochanter ridge area. During the operation, the blood supply of bone flap could be preliminarily analyzed by touching the pulsation of the branch artery, and the terminal branches of MFCA deep branches can be ligated and cut off at the far end of the branches. We therefore cut a piece of (4–5) cm × (1.5–2) cm × (1.5–2) cm bone flap with a special bone knife at the attachment of the medial circumflex femoral artery branch in the greater trochanter and inter-trochanter area behind the femur. Generally, two branch vessels can be reserved in the bone flap. The blood supply of the bone flap was re-evaluated through the bone flap cancellous bone bleeding. The vascular pedicle was partially released to enable the bone flap to transfer position, and the bone flap and its vascular pedicle were protected with saline-wet gauze (Fig. 1A, E). With the help of X-ray fluoroscopy, trephine drilling (Fig. 1D) was drilled into the articular surface of the weight-bearing area of the femoral head 3–5 mm below the cartilage (Fig. 1F, G), and the bone core was removed. The necrotic bone was removed with a grinding drill and curettage, and the bottom of the decompression channel was filed flat with a flat head file. Fresh free cancellous bone or artificial bone was properly implanted at and around the bottom of the decompression channel. The vascularized bone flap was implanted into the decompression groove in the neck of the femoral head, so that the cancellous bone of the bone flap was closely attached to the inner wall of the decompression channel with one side upward, and the cortical bone and vascularized bone flap was placed downward to avoid the distortion, compression or excessive pulling of the vascular pedicle (Fig. 1B, C). Finally, a 3.5-mm hollow screw was used to fix the screw, and pressure was applied appropriately.

#### Evaluation of postoperative efficacy

Harris hip score, anteroposterior and lateral hip radiographs and frog position hip radiographs were performed postoperatively, and SPECT/CT of femoral head flow imaging was used to evaluate the activity of bone graft flap and the recovery of blood supply to the necrotic area.

### Establishment and verification of finite element model

#### CT-based 3D reconstruction

Before operation, the hip was scanned with thin-slice CT with a slice thickness of 1 mm. Thereafter, the image was saved and exported in Dicom format, and the CT image of the left hip was modeled, and the acquisition of CT data was carried out after obtaining the informed consent of patients.

Mimics19.0 was used to build a 3D geological model in STL format for Dicom format images, and the STL file was imported into Geomagic Studio2014 to enable the surface fitting and smoothing. The femur structure model was imported corresponding to each group into Hypermesh14.0 software for mesh generation, and the file was exported in BDF format. Three-dimensional finite element models with 4-node isoparametric tetrahedral elements were used to mode the pelvis, plates and screws. An average element size of 0.6 mm was used for the cortical and cancellous bone, while for the screws element sizes were for 0.8 mm. A coefficient of friction of 0.1 was used for mechanical contact between the screw and femur. Thereafter, the finite element preprocessing software MSC.Patran2012 was imported to complete the finite element mesh attribute setting, boundary condition constraint, loading, material parameter definition and other steps. Finally, the calculation results were obtained in the finite element software MSC.Nastran2012. We next applied the same constraint conditions and load to the model of the upper segment of the normal femur and performed the finite element simulation to calculate the Von Mises and Min Principal equivalent stress cloud of the upper segment of the normal femur. It was then compared with the previous finite element studies to verify the efficacy of the femur structure model by adding physiological loads to observe the force distribution of the model [8] (Table 1).

**Table 1:** Material properties used for various components in the finite element model

Components	Young’s modulus (MPa)	Poisson’s ratio	Yield strength
Cortical bone	15,100	0.30	111
Cancellous bone	445	0.22	19.4
necrotic bone	124.6	0.15	5.5
artificial bone	334	0.30	1.85
Screw	105,000	0.30	800

**Fig. 2:**
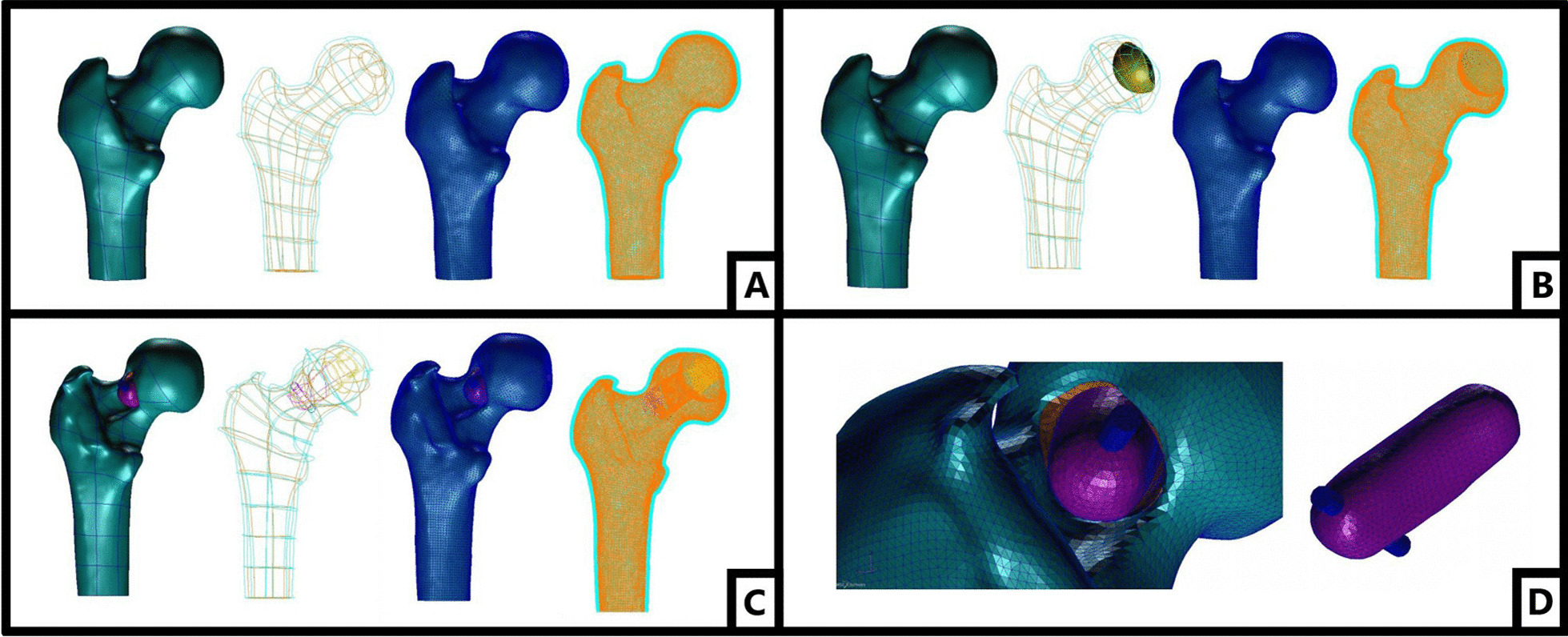
**A** The finite element model of normal group; **B** the finite element model of osteonecrosis group; **C** the finite element model of postoperative group; **D** the finite element model of bone flap in postoperative group

Osteonecrosis group: The shape of the femoral head necrotic area in the osteonecrosis group was reconstructed based on the actual situation of the affected hip. The volume of the necrotic area accounted for approximately 20–25% of the volume of the femoral head. A total of 65,247 nodes and 346,127 tetrahedral units were established by the model. (Fig. 2A).

Normal group: The upper femur contour was extracted before operation for replacement of the necrotic area with the normal bone tissue and establishment of the normal group finite element model. A total of 62,785 nodes and 341,240 tetrahedral units were established by the model (Fig. 2B).

Postoperative group: The postoperative group model was reconstructed according to the actual postoperative situation. Under ideal conditions, that all the necrotic focus has been cleared and filled with artificial bone. The size and shape of the bone flap and decompression passage were processed according to the actual image data. The top of the bone flap was placed 2 mm under the subchondral bone of the femoral head. The bone flap was set according to the actual situation. The upper part consisted of a cancellous bone that was in contact with the inner wall of decompression passage. The lower side connecting vascular pedicle was a cortical bone, which was not in contact with the inner wall of decompression passage. The lower part of the bone flap was fixed with a screw. A total of 63,424 nodes and 34,265 tetrahedral units were established by the model. (Fig 2C).

#### Mechanical analysis of efficacy and safety of modified MFCVBG in repairing ONFH

The boundary constraints were applied on each femur structure model. The lower end of the femur model was constrained and fixed to limit the degrees of freedom of each node in six distinct directions (three translations, three rotations). Referring to the previous studies [8], the hip joint reaction force was loaded on the surface of about 1.9 cm^2^ of the load-bearing area of the femoral head, that is, the loading range consisted of the elliptical area above the femoral head. Additionally, the loading direction of the iliotibial fasciculus muscle force was downward and parallel to the femoral shaft. The abductor muscle strength was loaded obliquely upward at an angle of about 29.5° with the femoral shaft. In view of the fact that the CT image of this study was obtained from an adult male weighing 72 kg, and the hip joint reaction load in this study was calculated according to the 70 kg (~ 700 N) human body weight (weight abbreviated as "G"). In addition, the force on the femoral structure of each group was analyzed under four different loads in the gait cycle of 0.5 times the body weight (0.5 G, standing on two feet), 2.75 G (standing on one foot), 4 G (walking with the middle foot on the ground) and 7 G (walking with the toe off the ground) [9]. Referring to the previous research data [8], it was assumed in this study that the iliotibial fasciculus muscle strength and abductor muscle strength were proportional to the hip joint reaction force under different loads.

#### Risk prediction of femoral head collapse risk

According to previous finite element studies of ONFH femoral head collapse [10], the risk of femoral head collapse was reflected by the collapse coefficient (structural stress/structural yield strength). We selected 0.1 as the physiological limit of necrotic bone tissue. The critical stress was 0.55 MPa as the yield strength of necrotic bone is 5.5 MPa and the stress index was 0.1. When there was less bone tissue found exceeding the critical stress, only stress compensated incomplete micro-fracture can occur. If the bone tissue exceeding the critical stress accounts for more than 50% of the total necrotic tissue, the large-scale collapse of the necrotic area might occur. In this study, the necrotic area bone tissue was selected for the collapse risk prediction and analysis in the necrosis group, and the bone tissue corresponding to the necrotic area was selected for analysis in the normal group and the postoperative group.

#### Simulation of the different healing conditions of postoperative bone flap

Based on the modified MFCVBG group, the different healing degrees of the inner wall of the bone flap and decompression passage were simulated to analyze the stress and displacement of the femoral head after loading during the healing and repair of the bone flap and decompression passage. Based on the previous fracture healing finite element studies [8], a model of different healing degrees of bone flap was established. Assuming that the healing degree was 0%, 25%, 50%, 75% or 100%, the stress distribution of the complete upper femoral segment as well as the femoral head, bone flap and screw, and the displacement under different loads (0.5G, 2.75G, 4G and 7G) of the whole femoral head and bone flap was analyzed. The different healing degrees were set in the calculation according to the following method: In the 100% healing state, the bone flap cancellous bone surface and the decompression passage inner wall cancellous bone are completely co-noded, that is, the original modified MFCVBG postoperative group. The 0% unhealed state indicated the immediate state after operation. The contact friction between the bone flap cancellous bone surface and the decompression passage inner wall cancellous bone was set, and the friction coefficient was 0.1. The sliding friction was allowed between the bone flap and the inner wall. 25% of the healing state was identified at the initial stage of healing. A circle of grid cells was taken from the inner wall of the decompression passage corresponding to the bone flap cancellous bone as the structure of the fusion zone. With reference to the previous studies [11] about the parameters involved in the process of fracture healing, the elastic modulus of granulation tissue at the initial stage of healing was 0.05 MPa, and the Poisson's ratio was 0.30. At 50% and 75% healing state, the structure of fusion zone was approximately selected as 50% or 75% of the material parameters of cancellous bone.

#### Postoperative model of different bone flap length, clearance degree of necrotic focus

It has been observed that during the actual operation, it is sometimes difficult to simultaneously place the long bone flap just below the subchondral bone, completely remove the necrotic bone tissue, and perform autologous bone as well as artificial bone filling. Therefore, in order to study the non-idealized state of modified medial femoral circumflex vascularized bone grafting such as short bone flap of trochanter or failure to complete the necrotic bone, this study established a short greater trochanter bone flap model based on the existing idealized the postoperative group model (indicated as short bone flap model, and the relative original postoperative group was referred to as long bone flap model), in which the necrotic area bone tissue was completely removed, the artificial bone was filled and placed under the artificial bone. It aided to establish decompression passage, however, it neither completely removed the necrotic bone tissue around the passage nor filled in the artificial bone, but only directly placed the greater trochanter bone flap into the incomplete cleared necrotic focus model (denied as incompletely cleared necrotic focus model). Additionally, the original ideal postoperative model could also be termed as a thorough elimination of the necrotic focus + long bone flap model. A total of 62,947 nodes and 33,986 tetrahedral units were established by the not completely clear + long bone flap model; a total of 62,579 nodes and 33,895 tetrahedral units were established by the not completely clear + short bone flap model.

## Results

### Evaluation of postoperative efficacy

**Fig. 3:**
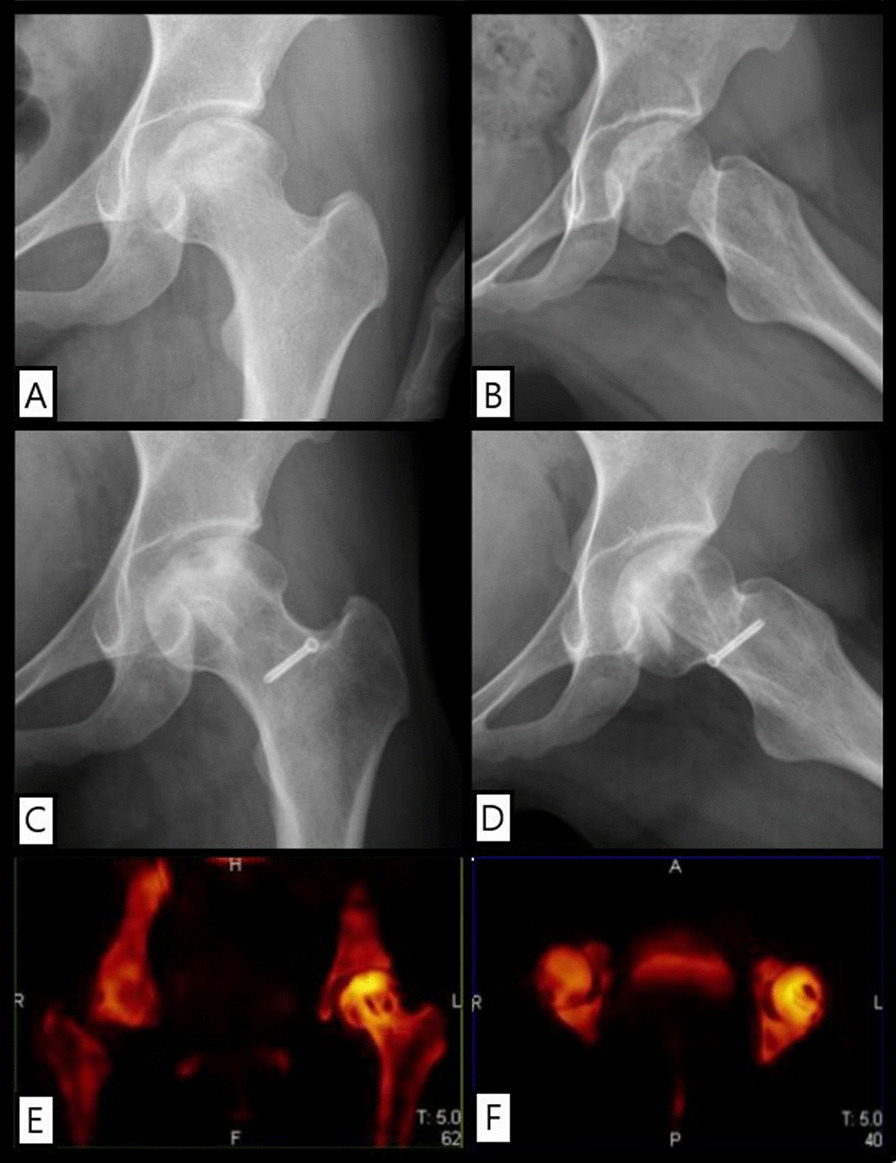
**A**, **B** Anteroposterior and lateral hip radiographs; **C**, **D** frog position hip radiographs and **E**, **F** SPECT/CT of femoral head flow imaging were performed one year after operation

One year after operation, HHS was improved to 81 points. During the follow-up period, the bone flap and the inner wall of decompression channel healed well, no osteonecrosis progression, no local collapse or micro-fracture occurred in the femoral head, and the articular surface was intact and the necrosis was well repaired. SPECT/CT suggested that the grafted bone flap was alive, and the original necrotic area around the flap was metabolically active. No obvious abnormality of bone metabolism was observed in the distribution area of the original MFCA terminal branch (Fig. 3).

### Verification of finite element model

In the gait cycle of the normal adults, the structural stress peak of the upper femur is found near the distal medial and lateral cortical bones, in which the medial part is primarily compressive stress and the lateral part is mainly tensile stress. In the upper femur model of this study, combined with Von Mises and Min Principal stress cloud, it was found that in the normal group, the maximum tensile stress peak of the femoral structure appeared at the distal lateral side of the model, about 29.7 MPa. The maximum compressive stress peak appeared at the far inner side of the model, about 84.5 MPa. The stress distribution morphology and peak value of femur were consistent with the results of the classical experimental mechanics research [12], which suggested that the proposed model was correct and effective. Based on this model, models of the necrosis group, the postoperative group and other groups can be established and finite element biomechanics research can be carried out in future studies.

### Mechanical analysis of the efficacy and safety of modified MFCVBG in repairing ONFH

We next analyzed the stress of three groups of femur models and found that in the gait cycle, with the load increasing from 0.5G to 7G, the cortical bone stress peak of normal femoral head ranged in 5.9–84.8 MPa. However, the cortical bone stress peak of necrotic femoral head ranged in 15.9–111.0 MPa, whereas the stress peak reached the yield strength under loads of 4G and 7G, but the cortical bone stress peak of postoperative femoral head ranged in 6.1–102.8 MPa. It was found that compared with the stress data of the normal group, the overall stress peak increment of the upper femur in the necrosis group under different loads was 3.7–145.0%, and increment of cortical bone stress on femoral head was 30.9–199.4%. The stress increment was the most significant in the state of 2.75G, while the stress peak at 7G increased slowly and remained near the structural yield strength. In addition, compared with the normal group, the overall stress peak of the upper femur and the cortical bone stress peak of the femoral head in the postoperative group increased slightly, with the increment of 2.1–5.9% and 3.4–21.2% (Table 2).

**Table 2:** Maximum Von Mises stress on femoral head in three groups

Loads	Normal group	Osteonecrosis group	Postoperative group
0.5G	5.9	15.9	6.1
2.75G	32.9	98.5	36.0
4G	48.1	111.0	54.8
7G	84.8	111.0	102.8

**Fig. 4:**
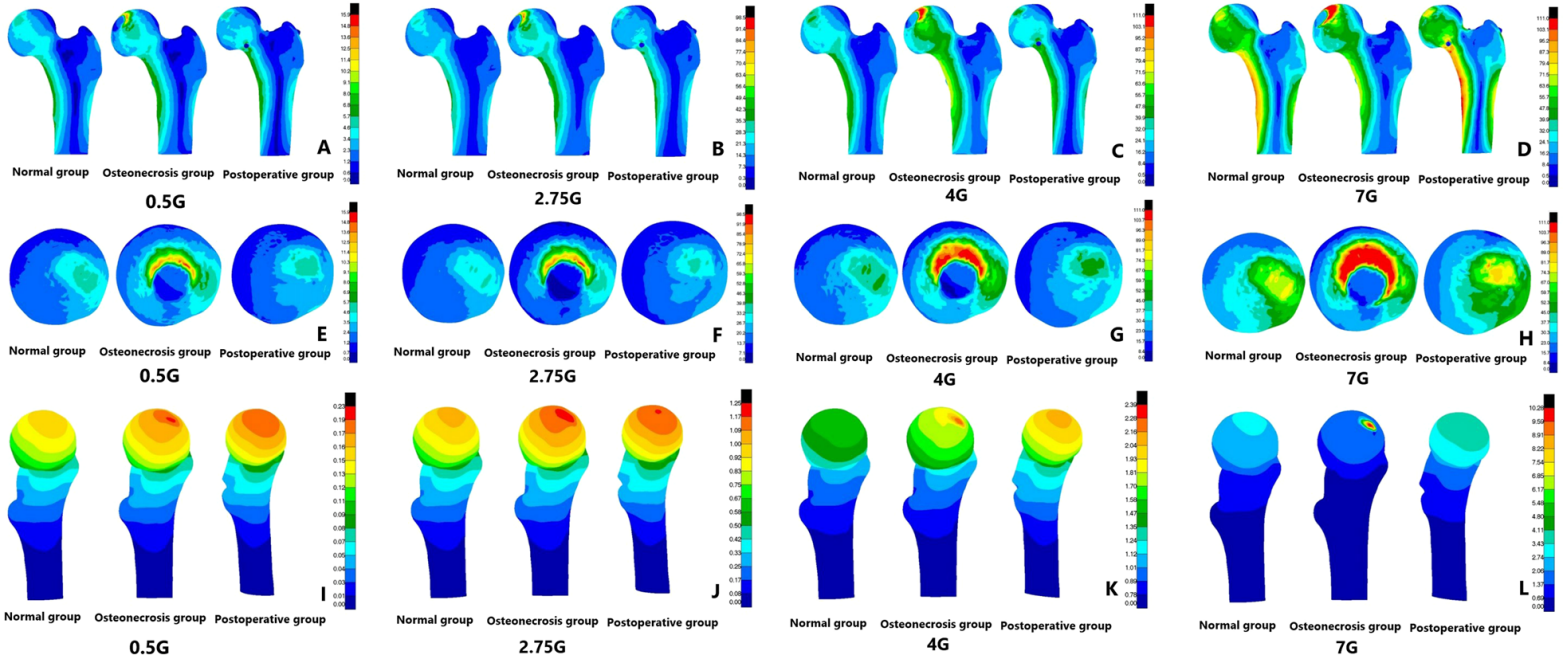
**A**–**D** the stress nephogram of proximal femur under 0.5G, 2.75G, 4G and 7G in anteroposterior view; **E**–**H** the stress nephogram of femoral head under 0.5G, 2.75G, 4G and 7G; **I**–**L** the stress nephogram of proximal femur under 0.5G, 2.75G, 4G and 7G in lateral view

**Fig. 5:**
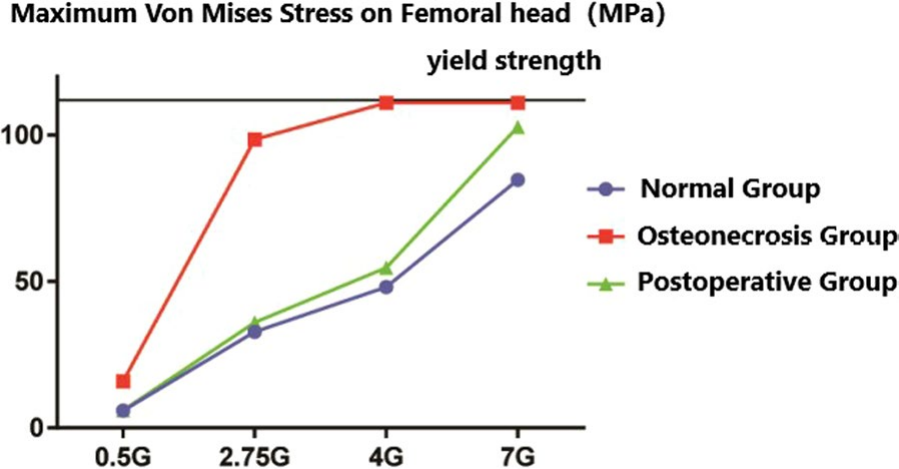
Maximum Von Mises stress on femoral head in three groups

**Fig. 6:**
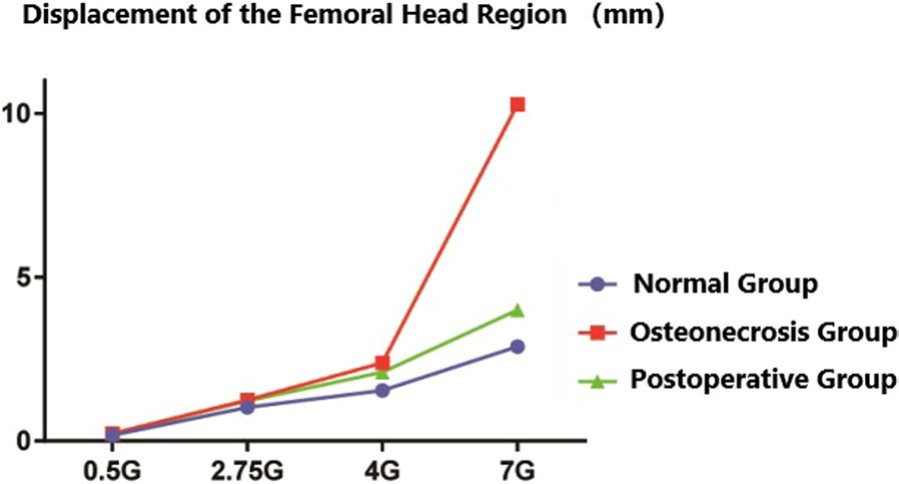
Displacement of the femoral head region in three groups

In the displacement cloud, the displacement peak of normal femoral head ranged in 0.18–2.88 mm, whereas the displacement peak of necrotic femoral head ranged in 0.23–10.28 mm. However, the displacements peak under loads of 4G and 7G was 2.39 and 10.28 mm, respectively, thus indicating a significant increase in displacement. The displacement peak of postoperative femoral head ranged in 0.22–4.00 mm. It was observed that compared with the normal group and the femoral head area, the displacement peak increment of the necrosis group was 21.4–256.9%, of which the displacement increased rapidly at the state of 7G, and the displacement peak increment of the postoperative group was 18.4–38.9% (Figs. 4, 5, 6).

### Mechanical analysis of different healing models of bone flap

**Fig. 7:**
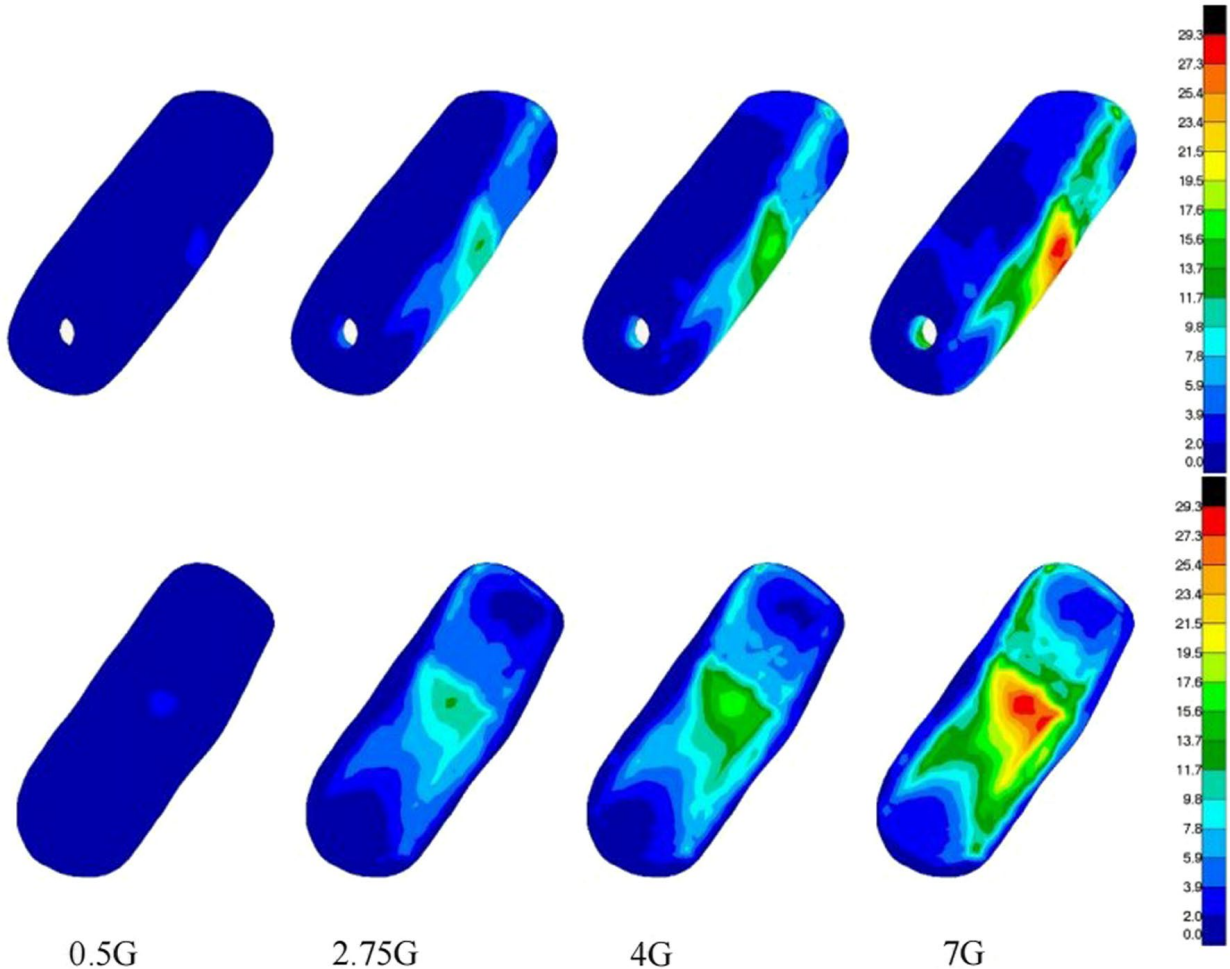
The stress nephogram of bone flap under 0.5G loads, 2.75G loads, 4G loads and 7G loads

**Fig. 8:**
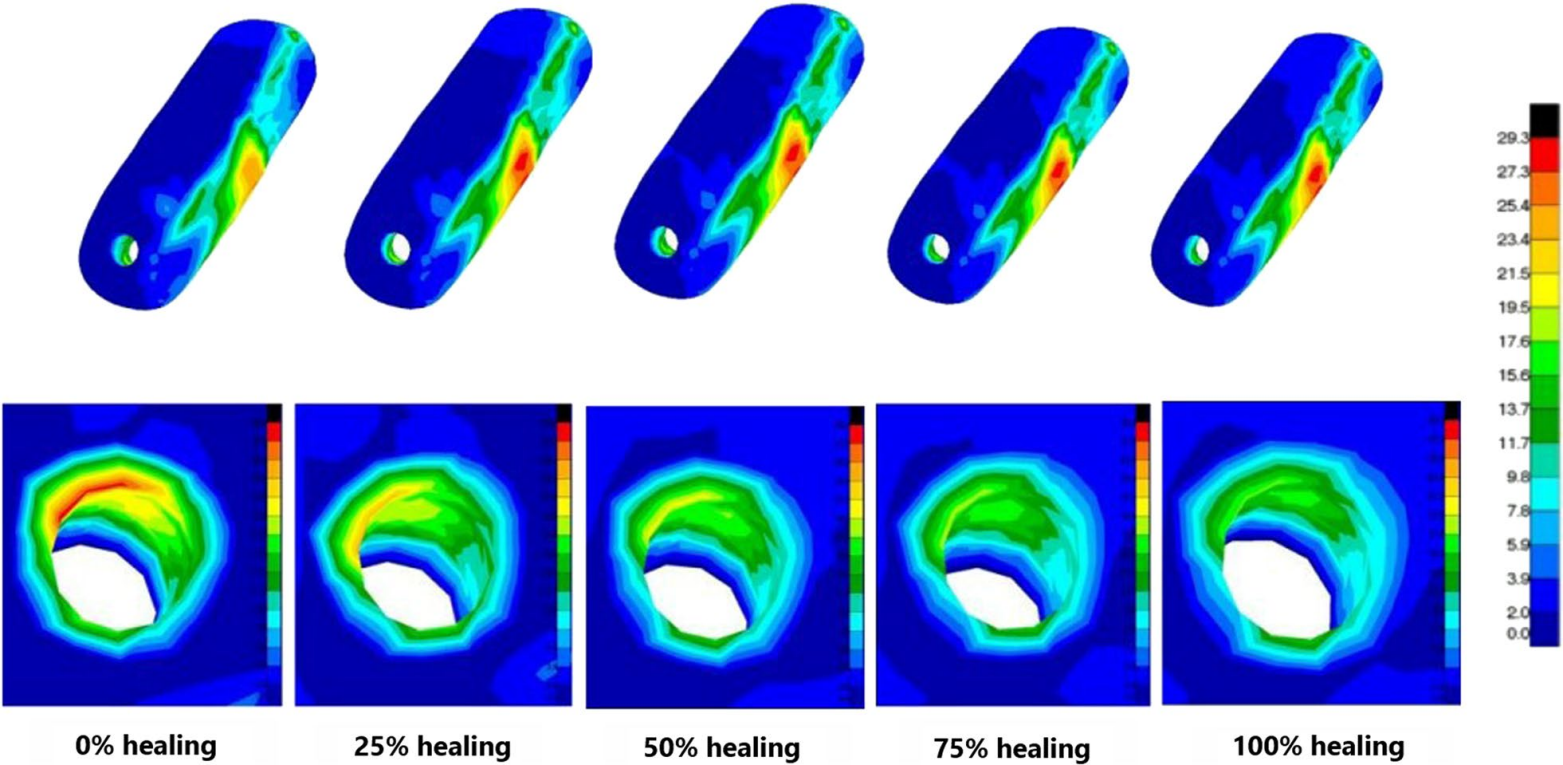
The stress nephogram of bone flap with different healing degrees under 7G loads

It was found that with the gradual healing of the inner wall of bone flap and decompression passage, the overall stress peak, cortical bone stress peak of femoral head, and displacement of femoral head of the upper femur decreased gradually. The bone flap stress peak increased gradually, but the overall maximum deformation, the stress around the screw and the bone flap screw hole decreased gradually. Under the different loads, it was noted that compared with the inner wall of the bone flap and decompression passage in the state of complete healing, the overall stress of the upper femur and the femoral head cortical bone stress peak increased immediately after the operation by 6.7–7.3% and 3.2–4.9%, respectively. The displacement of the femoral head increased slightly but was less than 5%. However, in the completely unhealed state, the stress on bone flap was less, but the stress on screw was greater, and the maximum stress peak increment was 5.2%. After complete healing, although the stress on the bone flap increased slightly, the overall deformation peak was relatively smaller, and the bone flap deformation peak changed within 5% (Fig. 7, 8; Table 3).

**Table 3:** Mean collapse coefficient under different loads in three groups

Loads	Normal group	Osteonecrosis group	Postoperative group
0.5G	0.00	0.01	0.00
2.75G	0.02	0.08	0.02
4G	0.02	0.13	0.03
7G	0.04	0.17	0.05

### Mechanical analysis and comparison of postoperative model with different bone flap length, clearance degree of necrotic focus

**Fig. 9:**
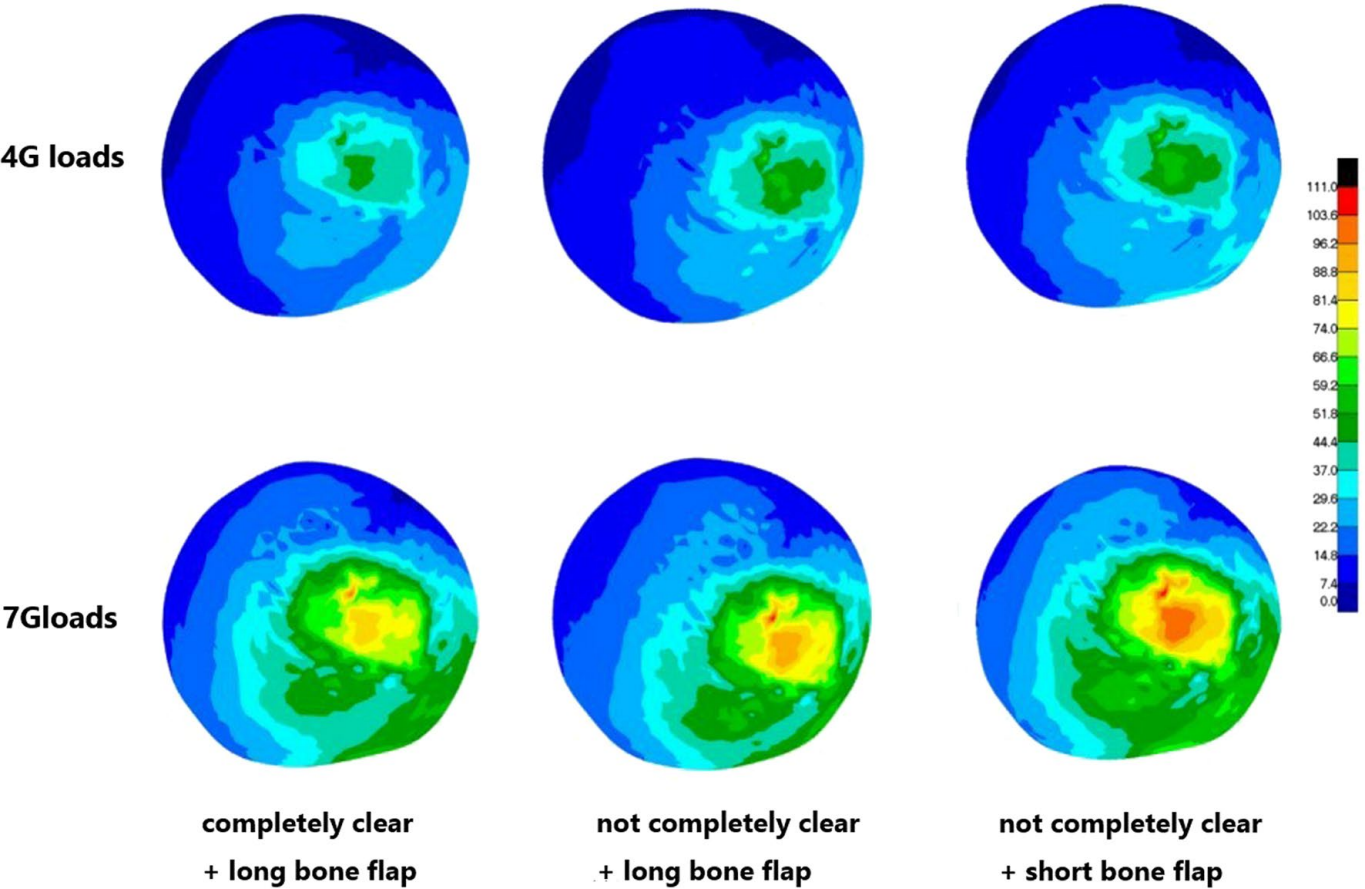
The stress nephogram of femoral head in different postoperative models under 4G loads and 7G loads

The mechanical analysis of the three postoperative models revealed several important issues from the perspective of structural stress. The two distinct groups of incomplete removal of necrotic focus + long bone flap model and the complete removal of necrotic focus + short bone flap model displayed larger stress peaks in the overall upper femur and femoral head region under loads of 4G and 7G upon comparison with the complete removal of necrotic focus + long bone flap model. Especially, the difference in the structural stress on femoral head was observed to be more significant. The maximum stress peak increments of the two groups were 14.6% and 15.7%, respectively. Indeed, the cortical bone stress peak of femoral head of the short bone flap group under load of 7G attained yield strength of the material. Mechanical analysis of the bone flap and screw revealed that compared with the complete removal of the necrotic focus + long bone flap model, the stress peak of model bone flap and screw in the two groups of non-ideal surgical models was significantly smaller, especially the difference in the bone flap stress peak. A reasonable explanation could be that the upper end of bone flap in the short bone flap group bone flap was lower, while the rigidity of the residual necrotic bone materials in the front end and surrounding area of the bone flap in the incomplete removal of necrotic focus was lower. In addition, the length of the bone flap in the short bone flap group was markedly decreased and the overall deformation of the bone flap decreased correspondingly. The necrotic focus of the long bone flap was completely removed, and the overall deformation of the bone flap was found to be similar in the two groups. The overall displacement of the bone flap was analyzed and average displacement of the bone flap in the non-ideal surgical model in the two different groups of incomplete removal of necrotic focus + long bone flap model and the complete removal of necrotic focus + short bone flap model increased significantly and their maximum average displacement increments were 8.6% and 9.7%, respectively. A reasonable explanation could be that the bone flap of the complete removal of necrotic focus + long bone flap group was directly placed at the bottom of the decompression passage, and the bone flap cortical bone can provide substantial mechanical support. However, in the two groups of non-ideal surgical procedures, the above and around of the bone flap were connected with the materials with relatively weaker rigidity and more easily deformed, so the bone flap exhibited a greater displacement and a tendency to slide downward (Fig. 9; Table 4).

**Table 4:** Maximum Von Mises stress on femoral head in different postoperative models under 4G loads and 7G loads

Loads	Completely clear + long bone flap	Not completely clear + long bone flap	Not completely clear + short bone flap
4G	45.8	62.8	63.4
7G	102.8	110.1	110.0

## Discussion

For young patients, the long-term complications associated early joint replacement surgery can often increase, the revision surgery is difficult, and the effect is not very ideal [13]. Filippo Migliorini et al. [14] shown that male gender, longer symptom duration before treatment, higher VAS scores, and lower HHS scores were negative prognostic factors after treatment for osteonecrosis of the femoral head. Therefore, Make a definite diagnosis as early as possible and the ONFH hip preservation treatment [15–17] of ONFH has become one of the primary research focus of ONFH, especially the hip preservation surgery with vascularized bone transplantation as the core technology [18]. Its aims to prevent the femoral head collapse in the short term after operation, block necrosis in the early stage of collapse, or lift up a slight collapse to optimally restore the morphology of femoral head, and maintain the blood supply of femoral head in the long term after operation, so as to achieve effective bone reconstitution of necrotic area. Herein, vascularized fibular graft [19] and quadratus femoris muscle pedicle bone grafting [20] are two main methods employed for vascularized bone grafting, each with its own distinct characteristics. It has been found that the former has a more accurate effect of hip preservation, but needs to sacrifice the fibula, which can be highly traumatic, and needs intraoperative microsurgical anastomosis of the blood vessels [21]. The latter is less invasive, does not need to anastomose blood vessels, and is relatively simple surgical procedure. However, the mechanical properties of the bone flap are not as good as the former, so it is often difficult to ensure the ideal position of the bone flap implantation [22]. The proposed modified MFCVBG belongs to a specific kind of quadratus femoris muscle pedicle bone grafting. In order to solve the significant problem that the bone flap can be difficult to be implanted in the ideal position. In 1943, the finite element method was first proposed by Courant et al.[23], whose basic principle is to approximate a continuum composed of infinite particles and infinite degrees of freedom as an aggregate composed of finite elements. Bae et al. [24] firstly applied the finite element method in the study of orthopedic biomechanics to explore the stress distribution in the femur. With the deepening of the research on osteonecrosis of the femoral head, the role of biomechanical factors in the treatment of osteonecrosis of the femoral head has been paid more and more attention. At present, it is widely believed that osteonecrosis of the femoral head is the result of the joint action of biology and mechanics [25]. This study used the finite element method to explore the mechanical safety of modified MFCVBG, to guide the implantation position of the bone flap during surgery and validate the biological mechanical stability of the modified MFCVBG.

For the hip preservation surgery of peri-collapse period ONFH, restoring the biomechanics stability of femoral head is extremely important for the prognosis of hip preservation [26]. For quadratus femoris muscle pedicle bone grafting, the length of the transplanted bone flap should be about long, the amount of bone should be about large, and the shape should be more regular. The large area of the cortical bone under the bone flap is more conducive to provide reliable mechanical support [27, 28]. Heiner et al. [29] found that the best position of the implant was on the upper side of the femur, so that it could contact and support the subchondral bone plate. With the rapid development of the surgical instruments and intraoperative monitoring equipment, in this research, we improve the surgical tools of MFCVBG and guide the bone flap can be conveniently as well as accurately placed directly under the subchondral bone and thus more closely fitted with the inner wall of decompression passage. Therefore, modified MFCVBG possesses certain technical advantages for the restoration of the mechanical structure of the peri-collapse period ONFH femoral head. The study performed mechanical analysis on the models of the normal group, the necrosis group and the postoperative groups under different loads. As the load increased, the overall stress, cortical bone stress of femoral head and displacement of femoral head in each group showed an increasing trend, but the stress and displacement of the postoperative group were found to be relatively less than that of the necrosis group and closer to that of the normal group. In addition, compared with the normal group, a large deformation occurred in some areas of the femoral head of the necrosis group during loading. Furthermore, the yield strength of the necrotic tissues was low, resulting in the occurrence of stress failure above the necrotic area, whereas the occurrence of the severe stress concentration around the stress failure area. When the material yield strength has reached at the cortical bone stress above the part of the necrotic area, it can then enter the plastic failure stage, and there are micro-fracture and collapse trends. The above results conclusively indicated that the modified MFCVBG could effectively restore the mechanical stability of the necrotic functional head.

Early postoperative activities after hip joint can play an important role in the rehabilitation after hip joint surgery [30]. In this study, under the same load conditions, as the inner walls of the bone flap and decompression passage gradually healed, the bone flap stress slightly increased within the safe stress range, whereas the stress around the screw and the bone flap nail holes gradually decreased. The dependence of the bone flap on the fixed screw in reconstructing the mechanical stability of the femoral head becomes smaller and smaller gradually, and the bone tissue risk near the cut of the screw decreased accordingly. However, there was not a great difference found between the bone flap and the overall mechanical parameters of the femur between the unhealed model immediately after the surgery and the complete healing model. Hence, we suggest that: During the early postoperative period, patients can try to reduce the load with crutches or walking aids and carry out appropriate flat activities to effectively promote the early postoperative recovery and the healing as well as the remodeling of bone flap under the safe stress conditions. However, it might be still necessary to be cautious about the weight-bearing activities after operation, especially for actions that increase loading on femoral head such as going upstairs and downstairs, squatting down and getting up.

It has been reported that more thorough removal of the necrotic tissues, better repair of the necrotic area and more stable mechanical support can serve as the guarantee to improve the success rate of treatment [31, 32]. Brown et al. [33] analyzed the protective effect of subchondral bone plate on cancellous bone disease below it by finite element analysis and pointed out that the degeneration degree of cancellous bone structure was the main influencing factor of femoral head collapse; Pengfei Wen et al. [34] showed that necrotic area had a significant influence on femoral head fracture collapse. However, there is no research on the relationship between the complete removal of the necrotic area and the effect of surgery. In this study, compared with the complete removal of the necrotic focus + long bone flap model, the stress of non-ideal model femoral head in the other two groups was observed to be significantly increased. For instance, under a load of 7G, the cortical bone stress of part of the femoral head of the short bone flap group reached the yield strength, with a micro-fracture trend to occur. The increase in stress on femoral head might be related to the decrease in bone flap stress in the two groups of non-ideal surgical models, that is, the support provided by the bone flap decreased, but relatively more stress was borne by the cortical bone on the surface of femoral head. In the necrotic focus model that is not completely removed, the area around the bone flap is connected to the necrotic bone tissue that is relatively weak in rigidity and prone to the deformation and displacement [35]. However, in the short bone flap model, the bone flap with the cortical bone that can provide greater mechanical support is not directly placed under the subchondral bone, and the subchondral bone is filled with artificial bone and can provide sufficient mechanical support. Therefore, the average displacement of bone flap in these two models was significantly higher than that of the postoperative model, and the bone flap exhibited a greater downward displacement trend. Therefore, for modified MFCVBG and other similar preservation therapy, placing the bone flap with the longer length and larger cortical bone area as close to the subchondral bone as possible [36], rather than under a large number of the free cancellous bones or artificial bones, might be a better strategy to restore the mechanical stability of the femoral head. At the same time, the placement of bone flap with vascular pedicle near the subchondral bone could also be helpful to restore the blood supply of the central necrotic area, which is conducive for both repair and reconstruction. Although the finite element analysis of the postoperative model without complete removal of necrotic focus indicated that the postoperative repair effect had effectively restored the mechanical stability of the femoral head, but more thorough removal of the necrotic bone tissue could improve the mechanical reconstruction effect of the femoral head after modified MFCVBG. Overall, it might also be more conducive for the healing of the bone flap and surrounding tissues and the repair of the necrotic area.

## Limitations

1. 3D finite element mechanical analysis is a theoretical analysis of the real mechanical conditions, a relatively simplified and idealized modeling and calculation process. This study did not employ relatively complex soft tissue materials for analysis.
2. In the actual situation, due to the individual differences among the different patients, surgical operation and other factors, there will be some variations observed in the mechanical properties of the different femoral heads. Therefore, the conclusions of this study can only be used as a reference for the related clinical problems.
3. The proposed model primarily defines the necrotic focus of the femoral head, and the conclusions obtained have certain limitations.

## Conclusions

1. In this research, the 3D finite element model of femur after modified MFCVBG was established for the first time, and on this basis, the models of different degrees of bone flap healing and femoral head micro-fracture were established, which provided a reference for modeling of other finite element research.
2. From the mechanical point of view, the modified MFCVBG for the treatment of ONFH is proved to be effective, safe and theoretically feasible.
3. Suggestions for the optimization of modified MFCVBG are proposed from the perspective of mechanics: Modified MFCVBG is suitable for patients with ARCO stage II and IIIA. Necrotic focus should be completely removed during the operation and the long bone flap should be placed directly under the subchondral bone. For patients with better bone healing ability, a more positive attitude can be taken to promote early postoperative weight-bearing.

## Data Availability

Data under study are available on request from the corresponding author, which are not publicly available due to privacy or ethical restrictions.

## Abbreviations

ONFH: Osteonecrosis of the femoral head
MFCVBG: Medial femoral circumflex vascularized bone grafting
HHS: Harris score
THA: Total hip arthroplasty
JIC: Japanese (Osteonecrosis) Investigation Committee
